# Early fibrinogen concentrate therapy for major haemorrhage in trauma (E-FIT 1): results from a UK multi-centre, randomised, double blind, placebo-controlled pilot trial

**DOI:** 10.1186/s13054-018-2086-x

**Published:** 2018-06-18

**Authors:** Nicola Curry, Claire Foley, Henna Wong, Ana Mora, Elinor Curnow, Agne Zarankaite, Renate Hodge, Valerie Hopkins, Alison Deary, James Ray, Phil Moss, Matthew J. Reed, Suzanne Kellett, Ross Davenport, Simon Stanworth

**Affiliations:** 1 Department of Haematology, Oxford Haemophilia & Thrombosis Centre, Churchill Hospital, Oxford University Hospitals NHS Trust, Oxford, UK; 2 NIHR BRC Blood Theme, Oxford University, Oxford, UK; 3 NHS Blood and Transplant Clinical Trials Unit, Cambridge, Bristol UK; 4 Nuffield Division of Clinical Laboratory Sciences, Radcliffe Department of Medicine, University of Oxford, John Radcliffe Hospital, Oxford, UK; 5 Department of Emergency Medicine, John Radcliffe Hospital, Oxford, UK; 6 Department of Emergency Medicine, St. George’s Hospital, London, UK; 7 Emergency Medicine Research Group Edinburgh (EMERGE), Royal Infirmary of Edinburgh, Edinburgh, UK; 8 Department of Anaesthetics, University Hospital Southampton NHS Foundation Trust, Southampton, UK; 9 Centre for Trauma Sciences, Blizard Institute, Queen Mary University of London, London, UK; 10 NHS Blood and Transplant, John Radcliffe Hospital, Oxford, UK

**Keywords:** Fibrinogen replacement therapy, Haemorrhagic shock, Multiple trauma, Cryoprecipitate, Transfusion

## Abstract

**Background:**

There is increasing interest in the timely administration of concentrated sources of fibrinogen to patients with major traumatic bleeding. Following evaluation of early cryoprecipitate in the CRYOSTAT 1 trial, we explored the use of fibrinogen concentrate, which may have advantages of more rapid administration in acute haemorrhage. The aims of this pragmatic study were to assess the feasibility of fibrinogen concentrate administration within 45 minutes of hospital admission and to quantify efficacy in maintaining fibrinogen levels ≥ 2 g/L during active haemorrhage.

**Methods:**

We conducted a blinded, randomised, placebo-controlled trial at five UK major trauma centres with adult trauma patients with active bleeding who required activation of the major haemorrhage protocol. Participants were randomised to standard major haemorrhage therapy plus 6 g of fibrinogen concentrate or placebo.

**Results:**

Twenty-seven of 39 participants (69%; 95% CI, 52–83%) across both arms received the study intervention within 45 minutes of admission. There was some evidence of a difference in the proportion of participants with fibrinogen levels ≥ 2 g/L between arms (*p* = 0.10). Fibrinogen levels in the fibrinogen concentrate (FgC) arm rose by a mean of 0.9 g/L (SD, 0.5) compared with a reduction of 0.2 g/L (SD, 0.5) in the placebo arm and were significantly higher in the FgC arm (*p* < 0.0001) at 2 hours. Fibrinogen levels were not different at day 7. Transfusion use and thromboembolic events were similar between arms. All-cause mortality at 28 days was 35.5% (95% CI, 23.8–50.8%) overall, with no difference between arms.

**Conclusions:**

In this trial, early delivery of fibrinogen concentrate within 45 minutes of admission was not feasible. Although evidence points to a key role for fibrinogen in the treatment of major bleeding, researchers need to recognise the challenges of timely delivery in the emergency setting. Future studies must explore barriers to rapid fibrinogen therapy, focusing on methods to reduce time to randomisation, using ‘off-the-shelf’ fibrinogen therapies (such as extended shelf-life cryoprecipitate held in the emergency department or fibrinogen concentrates with very rapid reconstitution times) and limiting the need for coagulation test-based transfusion triggers.

**Trial registration:**

ISRCTN67540073. Registered on 5 August 2015.

## Background

Uncontrolled bleeding is the most common preventable cause of death in major trauma [1]; it affects up to 40% of patients with severe injury. Trauma haemorrhage is exacerbated by a complex interplay of clotting abnormalities and may result in a trauma-induced coagulopathy (TIC). Activation of protein C is central to TIC [2] and associated with increased fibrinolysis [3] and loss of fibrinogen [4]. Hypofibrinogenaemia occurs early after injury and is an independent predictor of death [5]. Augmentation of blood fibrinogen levels necessitates the use of a concentrated form of fibrinogen supplementation (e.g., cryoprecipitate or fibrinogen concentrate [FgC]) because fresh frozen plasma (FFP) alone is ineffective [4, 6].

There is continued debate about the comparative effectiveness of the two concentrated fibrinogen treatments [7, 8], despite two recent feasibility randomised controlled trials (RCTs) in trauma haemorrhage (CRYOSTAT 1 [9] and FiiRST [10]). The aim of this paper is to report the findings of a second feasibility study in adult trauma haemorrhage using FgC. The primary objectives of the E-FIT 1 study were to determine whether it was possible to deliver FgC therapy early (within 45 minutes) to adult trauma patients and the proportion of participants whose fibrinogen levels were maintained ≥ 2 g/L during active haemorrhage.

## Methods

### Study design E-FIT 1 trial

The E-FIT 1 study was a multi-centre, double-blind, placebo-controlled RCT conducted in five UK major trauma centres. The study is registered with www.controlled-trials.com (ISRCTN67540073).

### Eligibility criteria and randomisation

Trauma patients were eligible if they were adults (judged to be aged 16 years or older), were actively bleeding and in haemorrhagic shock and therefore required activation of the major haemorrhage protocol (MHP) or had already received a transfusion of emergency red blood cells (RBC). Exclusion criteria included patient transferred from another hospital, the trauma team leader deemed the injury incompatible with life, more than 3 hours had elapsed from time of injury, pregnant women and severe isolated or unsalvageable head injury. Women of childbearing age had a point-of-care blood test performed to rule out pregnancy (Abbott, Princeton, NJ, USA).

Participants were block randomised in a 1:1 ratio of placebo to active arm. An independent statistician produced the allocation sequence using a computer-generated random sequence, and randomisation lists were produced centrally. Allocation concealment was maintained by the labelling of study packs prior to release to sites. Identical study packs containing either FgC or placebo were sequentially labelled, and participants were allocated to the next available study pack. All research site staff, participants and trial management staff were blinded to study allocation.

### Consent

An emergency waiver with independent agreement process was used. Written informed consent from the participant was sought as soon as practically possible after study entry for continuation in the trial. If the participant did not regain capacity, agreement was sought from the participant’s next of kin or other appropriate representative. The protocol and consent process was approved by the NHS National Research Ethics Service (NRES) Committee South Central Oxford C Ethics Committee (15/S3/0316) and the Medicines and Healthcare products Regulatory Agency (MHRA) (25224/0003/001-0001).

### Study intervention

Participants were randomised equally to treatment or placebo. All randomised participants received MHP, and the study intervention was started as soon as possible and within 45 minutes of hospital arrival. Typically an MHP constituted two transfusion packs—pack 1 followed by repeated use of pack 2—until bleeding was controlled. Pack 1 included 4 RBC and 4 FFP; pack 2 included 4 RBC, 4 FFP, 10 U of cryoprecipitate (approximately 300 ml, 4 g of fibrinogen) and 1 pool platelets. (Specifications for blood components are found in the UK ‘red book’ [11]). Standard laboratory clotting tests were taken throughout active bleeding, and a fibrinogen level of < 1.5 g/L was the trigger for additional cryoprecipitate, when necessary.

The dose of FgC was chosen using data from the CRYOSTAT 1 trial [9]. In this trial, two pools of UK cryoprecipitate, containing approximately 4 g of fibrinogen, constituted the study intervention, and fibrinogen levels were maintained above 1.8 g/L during active bleeding. Modelling data from a multi-centre European study have demonstrated that a fibrinogen level of 2.3 g/L is associated with the lowest mortality rates [12]. A dose of 6 g of FgC was therefore chosen because 2 g of fibrinogen raises the blood level in patients with major bleeding by approximately 0.5 g/L [13].

In the treatment arm, an infusion of 6 g of FgC (RiaSTAP; CSL Behring, King of Prussia, PA, USA) was administered as soon as possible, and an equivalent volume (300 ml) 0.9% saline was administered in the placebo arm. FgC and placebo were blinded interventions. Study intervention packs were held in the emergency department (ED). Blinding was maintained by research staff following a validated protocol [14, 15]. Reconstitution of the study intervention was completed at the patient’s bedside by research staff. The intervention drug was drawn into black syringes and infused as an intravenous bolus over 5 minutes. Participants were deemed to have received the study intervention if at least five whole syringes were infused.

### Outcomes

The primary outcome was the feasibility of administering the study intervention within 45 minutes of admission, defined by the proportion of all participants randomised who started their infusion within that time. For this trial to be successful, at least 90% of the participants were required to achieve this target. An additional primary outcome was to determine the proportion of participants whose fibrinogen level remained at 2 g/L or above during active haemorrhage.

Secondary outcome measures were clinical and laboratory measures of efficacy and safety. Clinical outcomes included mortality at 3, 6 and 24 hours, and 28 days from admission; transfusion requirements, in numbers of units, at 3, 6 and 24 hours; duration of organ support; in-patient stay, including the intensive care unit/high-dependency unit; and quality of life. Safety was measured by symptomatic thrombotic events and arterial (e.g., myocardial infarction, stroke) and venous (e.g., pulmonary embolism, deep venous thrombosis) events during hospital stay. Laboratory measures included Clauss fibrinogen level at 2 hours after admission and at day 7 from admission. Standard MHP protocols in the UK recommend frequent blood samples every 30–60 minutes during trauma haemorrhage [16] and were taken as standard measurements.

### Sample and data collection

Blood samples were drawn immediately upon admission to the resuscitation room. Clauss fibrinogen measures were analysed in the hospital coagulation laboratories, according to standard operating procedures. Patient characteristics, mechanism and severity of injury, and admission physiology were collected and scored using the Abbreviated Injury Scale, Injury Severity Score and Glasgow Coma Scale. Tranexamic acid and other haemostatic drug administrations were recorded. Organ support was defined using the composite time to complete organ failure resolution score [17]. Organ failure was defined using the Sequential Organ Failure Assessment score. Data on timing of transfusions, clear fluids and mortality were collected in the first 24 hours. Measures to mitigate venous thromboembolic risk were recorded weekly to day 28. Symptomatic thromboembolic disease was categorised according to standard clinical and/or radiological measures. Quality of life (EQ-5D-5L) questionnaires were completed upon discharge or day 28, whichever was the sooner. The EQ-5D-5L descriptive systems index value was calculated, allowing the five health dimensions to be converted into a single numeric measure [18].

### Sample size and data analysis

If the proportion of participants who received the study intervention within 45 minutes from admission was 90%, a sample size of 40 would yield a 95% CI for this estimate of between 76% and 97%. To allow for 20% drop-out, the final sample size was chosen to be 48 participants in total, with 24 participants per arm. All analyses were performed according to the intention-to-treat principle and included all randomised participants.

Clinical and laboratory measures were compared using Fisher’s exact test or the Mann-Whitney test for categorical or continuous data, as appropriate. Normal linear regression was used to assess whether fibrinogen at 2 hours from admission was different between the two arms, adjusting for values at admission. Normal linear regression, unadjusted for any other factors, was used to assess whether fibrinogen at 7 days from admission was different between arms. Residual plots from each normal linear regression were examined for evidence of non-linearity, skew or non-constant variance. Log-transformed fibrinogen would be used in the regression model(s) if any evidence was found. All-cause mortality was estimated using the Kaplan-Meier method and compared using the log-rank test. Duration of organ support and hospital stay were estimated using competing risks methods and compared using Gray’s test. Death prior to the event of interest was considered the competing risk.

Sensitivity analyses were conducted for the primary outcomes, treating cases with missing time of administration of the study intervention as administration within 45 minutes (best-case scenario) or as administration beyond 45 minutes (worst-case scenario) and treating cases with missing fibrinogen at 2 hours from admission as achieving (best-case scenario) or not achieving (worst-case scenario) a level of at least 2 g/L. Except where specified, all analyses were unadjusted, and there was no adjustment for multiple testing. All statistical tests were two-sided. All analyses were undertaken using SAS/STAT software version 9.4 (SAS Institute Inc., Cary, NC, USA).

An independent data monitoring committee monitored all safety events throughout the study. All serious adverse events were evaluated and classified independently by the co-chief investigators, and any disagreements were resolved by consensus. The study manuscript was produced according to Consolidated Standards of Reporting Trials (CONSORT) recommendations for the reporting of randomised clinical trials [19].

## Results

### Recruitment and baseline characteristics

**Fig. 1:**
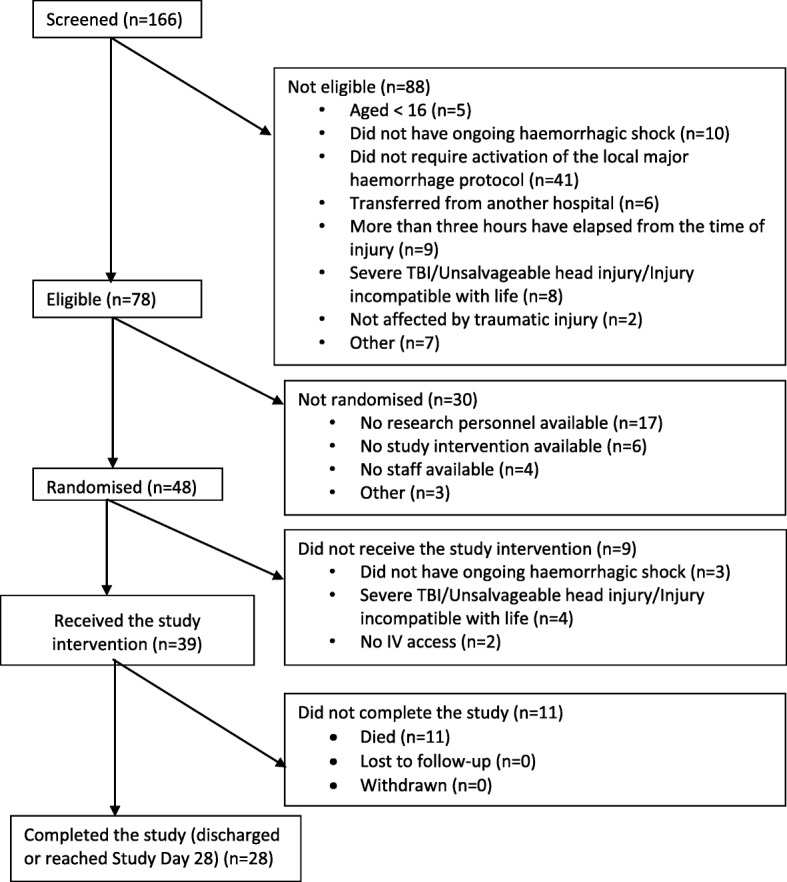
Consolidated Standards of Reporting Trials (CONSORT) flow diagram. Overall recruitment rate was 62% and ranged between 22% and 100% across the five centres. *TBI* Traumatic brain injury.

Of 166 adult trauma patients with major trauma haemorrhage who were admitted and screened between January 2016 and November 2016, 78 met eligibility criteria and 48 were randomised to the trial. The main reasons for ineligibility are set out in the CONSORT flow diagram (Fig. 1). Of the 48 participants randomised, 39 received study intervention. Of the nine who did not receive study intervention, no intravenous access could be established in two participants, and seven participants initially deemed eligible were subsequently found not to meet eligibility criteria: Three had no ongoing haemorrhagic shock (stabilisation of blood pressure and heart rate), and four were found to have unsalvageable traumatic brain injury. No participant withdrew consent or was lost to follow-up. One participant was screened and found to be ineligible owing to concerns regarding potential pregnancy.

Baseline characteristics were similar in the two study arms (Table 1), although participants in the FgC arm had a lower systolic blood pressure (86 mmHg vs. 95 mmHg), higher injury severity (34 vs. 29), and lower EXTEM clot amplitude at 5 minutes (26 mm vs. 35 mm) on admission. All participants received a 1-g bolus of tranexamic acid either pre-admission or upon arrival to hospital. Mean admission fibrinogen level was 1.6 g/L (SD, 0.7) in the FgC arm and 2.1 g/L (SD, 0.9) in the placebo arm (Table 2). No participant received platelets, cryoprecipitate or colloid pre-admission.

**Table 1:** Baseline characteristics

	Fibrinogen concentrate arm	Placebo arm
Subjects		
No.	24	24
Age, yr	38 (31–47)	36 (22–56)
Male, *n* (%)	20 (83)	19 (79)
Timelines		
Injury to hospital^a^, min	98 (77–118)	87 (66–116)
Injuries and admission physiologic measures		
Blunt	21 (88)	18 (75)
ISS	34 (24–43)	29 (22–34)
Systolic blood pressure, mmHg	86 (72–124)	95 (82–128)
Heart rate, beats/min	101 (88–116)	112 (93–126)
GCS	3 (3–14)	3 (3–15)
Clauss fibrinogen level, g/L	1.9 (0.9–2.2)	2.3 (1.6–2.5)
EXTEM CA5	26 (15–28)	35 (26–42)
FIBTEM CA5	4 (3–7)	7 (4–12)
Pre-randomisation		
TXA administered pre-admission	18 (75)	20 (83)
RBC, units	1 (0–2)	1 (0–2)
FFP, units	0 (0–1)	0 (0–2)
Crystalloids, ml	0 (0–475)	0 (0–625)

*Abbreviations: CA5* Clot amplitude at 5 min, *FFP* Fresh frozen plasma, *GCS* Glasgow Coma Scale, *ISS* Injury Severity Score, *RBC* Red blood cells, *TXA* Tranexamic acid

Data are number (%) for categorical variables and median (IQR) for continuous variables

^a^One participant was admitted to hospital > 3 h after injury (subsequently defined as a protocol deviation)

**Table 2:** Fibrinogen levels over time, by treatment arm

Outcome	Fibrinogen concentrate arm (*n* = 24)	Placebo arm (*n* = 24)	Overall (*n* = 48)	*p* Value
Fibrinogen, mean (SD)				
At admission	1.6 (0.7)	2.1 (0.9)	1.9 (0.8)	n/a
At 2 h from admission during first active haemorrhage^a^	2.8 (1.3)	1.8 (0.6)	2.3 (1.1)	< 0.0001
7 days from admission	6.7 (1.8)	7.5 (1.9)	7.1 (1.9)	0.2843

^a^*P* value adjusted for value at admission

### Primary outcomes

Twenty-seven of 39 participants (69%; 95% CI, CI 52–83%) across both arms received study intervention within 45 minutes of admission. It was not feasible to deliver study intervention within 45 minutes of hospital admission, and the pre-defined target of 90% compliance was not met. The median time to delivery of study intervention was 39 minutes (IQR, 28.0–54.0) across all participants and was similar in each arm (*p* = 0.56); in the FgC arm it was 37.5 minutes (IQR, 31.0–43.5), and in the placebo arm it was 40.0 minutes (IQR, 23.0–76.0). Twelve participants did not receive study intervention within 45 minutes, for the following reasons: awaiting pregnancy results (*n* = 2), inability to administer treatment whilst participant was in computed tomography scanner (*n* = 4), lack of intravenous access (n = 2), transfusion not commenced immediately upon admission (*n* = 3), and clerical error regarding the time when a participant was booked into the ED (*n* = 1).

Seventy-five percent (95% CI, 51–91%) of participants in the FgC arm (15 of 20 participants) had a fibrinogen blood level greater than or equal to 2 g/L during the first 2 hours of admission compared with 47% (95% CI, 23–72%) in the placebo arm (8 of 17 participants) (*p* = 0.10). The levels changed over time, as shown in Table 2. The mean fibrinogen level was higher in the FgC arm than in the placebo arm (*p* < 0.0001) at 2 hours from admission during active haemorrhage. In the placebo group, the fibrinogen level fell by 0.2 g/L (SD, 0.5), compared with a rise of 0.9 g/L (SD, 0.5) in the active arm. Sensitivity analyses of primary outcome measures revealed that the results presented were not sensitive to the missing data. There was no evidence of a difference in Clauss fibrinogen levels at day 7 between arms (*p* = 0.28). There was no evidence of non-linearity, skew or non-constant variance in any of the normal linear regression residual plots.

### Secondary outcomes including safety

**Fig. 2:**
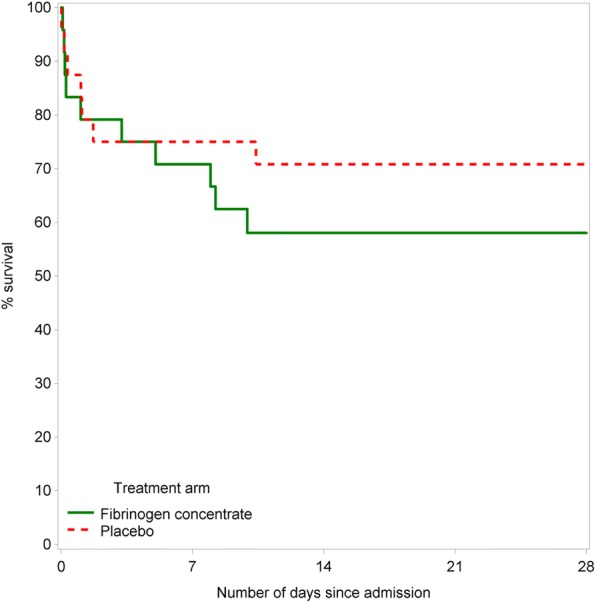
Survival to day 28, by treatment arm

There was no difference in transfusion requirements between arms (Table 3) in the first 24 hours for RBC, FFP and platelets, but we observed a trend towards more cryoprecipitate use in the FgC arm at 24 hours (*p* = 0.06). There were ten deaths in the FgC arm and seven in the placebo arm (Fig. 2), and six participants died prior to receipt of study intervention (two in the FgC arm and four in the placebo arm). All-cause mortality at 28 days was 35.5% (95% CI, 23.8–50.8%) overall, 42.0% (95% CI, 25.2–64.0%) in the FgC arm and 29.2% (95% CI, 15.1–51.6%) in the placebo arm. Of the 11 participants who died after receiving the study intervention, three died of uncontrolled bleeding (two FgC), five died of multiple organ failure (four FgC), one died of single organ failure (FgC), one died of traumatic brain injury (FgC) and one died of polytrauma (placebo). The times to death for the participants who died of haemorrhage were 1.9 and 4.9 hours (FgC) and 8.1 hours (placebo).

**Table 3:** Transfusion requirements during the first 24 hours

	Fibrinogen concentrate arm (*n* = 24)	Placebo arm (*n* = 24)	*p* Value
Units at 3 h			
RBC	4 (2–6)	2 (2–6)	0.73
FFP	3 (2–6)	3 (0–7)	0.92
Platelets	0 (0–1)	0 (0–1)	0.98
Cryoprecipitate	0 (0–2)	0 (0–1)	0.46
Units at 6 h			
RBC	3 (2–6)	2 (2–5)	0.62
FFP	4 (2–6)	3 (0–7)	0.77
Platelets	0 (0–1)	0 (0–1)	0.85
Cryoprecipitate	0 (0–2)	0 (0–0)	0.12
Units at 24 h			
RBC	4 (2–8)	2 (2–5)	0.38
FFP	5 (2–8)	3 (0–6)	0.39
Platelets	1 (0–1)	0 (0–1)	0.59
Cryoprecipitate	2 (0–2)	0 (0–0)	0.06

*FFP* Fresh frozen plasma, *RBC* Red blood cells

Data are median (IQR). At each time point blood component use was analysed for patients who were still alive within the specified time frame. One participant died within 2 h (± 30 min) of admission, two within 3 h (± 30 min) of admission, five within 6 h (± 1 h) of admission and seven within 24 h (± 4 h) of admission

We did not observe any difference in duration of organ support (median 17 days in both arms, *p* = 0.63), overall hospital length of stay (lower quartile 27 and 18 days [FgC vs. placebo], *p* = 0.49) or quality of life (median self-evaluated health scores, 48 and 55 [FgC vs. placebo], *p* = 0.39; median index values, 0.16 and 0.21 [FgC vs. placebo], *p* = 0.92). Eighty-three percent of participants (39 of 47) received venous thromboembolism prevention therapy by day 7 of their hospital admission, rising to 100% by day 28 (15 of 15). Serious adverse events are described in Table 4. Five patients experienced a thromboembolic event; two of the three arterial events occurred in the placebo arm, and two pulmonary embolic events occurred in the FgC arm.

**Table 4:** Serious adverse events

	Fibrinogen concentrate arm	Placebo arm
Subjects		
Number of participants in receipt of the study intervention	20	19
Number of participants experiencing at least one SAE^a^	13	11
Number of SAEs	29	21
Symptomatic thrombotic events	3	2
Arterial		
MI	0	0
Stroke	1	1
Other (arterial thrombus)	0	1
Venous		
DVT	0	0
PE	2	0
Sepsis	4	6
Organ failure	10	2
Multiple organ failure	4	1
Single organ failure	6	1
New-onset major bleeding	1	3
Uncontrolled major bleeding^b^	2	1
Other SAEs	9	7
Death		
All deaths^c^	8	3
Death due to bleeding	2 (25%)	1 (33%)

*Abbreviations: DVT* Deep venous thrombosis, *MI* Myocardial infarction, *PE* Pulmonary embolus, *SAE* Serious adverse event

Safety data were collected for only the 39 participants who were administered the study intervention

^a^Eleven participants experienced more than one SAE

^b^Major bleeding that was not controlled at any time from admission

^c^Includes all cases of multi-organ failure, all cases of uncontrolled bleeding, one case of single organ failure in the active treatment arm and two other SAEs (one in the active treatment arm and one in the placebo arm)

## Discussion

This is the second RCT evaluating administration of FgC in trauma. Despite median administration times approximating 40 minutes, the range varied widely (10 to 82 minutes), and only 69% of participants received study intervention within 45 minutes of admission. There was some evidence of a difference in the proportion of participants with fibrinogen levels ≥ 2 g/L between arms (*p* = 0.10). However, the FgC group had a fibrinogen level of 1.6 g/L on admission (0.7 g/L lower than the placebo arm), which likely reflects the trends towards higher injury severity and greater degree of shock in the intervention arm. Importantly, the fibrinogen level in the FgC arm rose by a mean of 0.9 g/L (SD, 0.5) compared with a reduction of 0.2 g/L (SD, 0.5) in the placebo arm, and average fibrinogen level at 2 hours from admission was significantly higher in the FgC arm (*p* < 0.0001). Fibrinogen levels did not remain elevated by 7 days, a finding also shown in the two other feasibility RCTs [9, 10], suggesting no long-term effect of fibrinogen replacement.

Transfusion requirements were not different between arms; however, it was notable that there was a trend towards increased cryoprecipitate use in the FgC arm. Transfusion needs were greater for all blood components in the FgC arm, again a difference likely reflecting the higher injury burden and shock, the empiric nature of transfusion administration, and the lower admission level of fibrinogen. The proportion of patients achieving haemostasis at 3 hours, however, was identical in both arms, suggesting that duration of bleeding does not fully explain the need for greater cryoprecipitate use in the FgC arm (data not shown).

No safety signal for thrombotic events was detected in this study. Venous thromboembolism was reported only in the FgC arm and was under 10%, a rate similar to that in a recent FgC trauma study in which researchers also used 6 g of FgC as their intervention [10]. More deaths were recorded in the FgC arm (*n* = 8 vs. *n* = 3), with no difference in deaths resulting from bleeding between arms, although the study was not powered for mortality. Differences in death rates may represent the variability in baseline characteristics between groups (i.e., higher injury severity and worse organ failure in the FgC arm).

Rapid reconstitution is often cited as an important benefit of FgC. Our study, and that of Nascimento et al. [10], reported similar median FgC reconstitution times: 23 minutes (SD, 9) (E-FIT1) and 26 minutes (SD, 5) (FiiRST). In a non-RCT setting, dissolution of fibrinogen for in vitro testing has been reported within 30 seconds [20], although it most commonly takes 10 minutes [21]. The longer reconstitution times in our study were due to the need to dissolve the fibrinogen whilst maintaining allocation concealment. The study vials were kept in cardboard tamper-proof boxes, meaning that dissolution of fibrinogen powder (or placebo) could not be visually assessed and that each vial needed to ‘rest’ for 3 minutes prior to being drawn into a syringe [14]. In an unblinded RCT, time to factor concentrate administration was shorter at 10 minutes (IQR, 10–16) [22]. A future alternative source of concentrated fibrinogen may be extended shelf-life cryoprecipitate [23], which could be pre-thawed and held in the ED, avoiding reconstitution times, but this has yet to be tested in an RCT setting.

The inclusion criteria for this trial did not use fibrinogen level. Goal-directed transfusion therapy using viscoelastic haemostatic assays (VHAs) (thromboelastography or rotational thromboelastometry [ROTEM]) is standard practice in many trauma centres in Europe and the United States [22, 24, 25], and it is advocated for guiding transfusion therapy. In a retrospective European study, VHA-guided therapy has led to a 50% reduction in the incidence of massive transfusions for trauma compared with that predicted by the trauma-associated severe haemorrhage score, with an associated reduction in mortality from 33% to 22% [26]. However, delays to first results are incurred (on average, 15–20 min [21, 22]), and empiric, immediate transfusion therapy for patients with uncontrolled haemorrhage is a standard of care [27]. To date, in trauma haemorrhage, there are no evidence-based thresholds to guide fibrinogen treatment, only expert consensus [28]. A small RCT, which was terminated early, used dual ROTEM measures to guide transfusion (FIBTEM A10 > 8 mm, EXTEM CT < 78 seconds), and these data may support the use of ROTEM to direct transfusion therapy [22] but need validation in a larger study. A large European study is due to be completed in 2018 (iTACTIC; ClinicalTrials.gov, NCT02593877) which will compare the efficacy of VHA and standard clotting tests for transfusion in trauma.

Our study was designed to include participants with the clinical phenotype of severe bleeding, not those exclusively with a low fibrinogen level. We did not use VHA or Clauss fibrinogen for two reasons: (1) to minimise delay to randomisation (VHA testing incurs 10–15-min delays) when speed of delivery of transfusion is known to be important [27, 29, 30] and (2) to include participants with the broader clinical entity of bleeding. The recent RETIC trial used FIBTEM A10 for eligibility, and randomisation took approximately 35–38 minutes, compared with 15–16 minutes in our study [22].

This is one of the first published multi-centre UK trauma transfusion studies, and it provides evidence that the delivery and conduct of RCTs in the challenging environment of the ED is possible. Prior to study start it was predicted that the recruitment would take 18 months. Recruitment was completed within 10 months, facilitated both by the research infrastructure and by the ability of research teams to recruit outside core hours. Rapid recruitment has recently been seen in another transfusion trauma study (FEISTY; ClinicalTrials.gov, NCT02745041) showing an encouraging appetite for the collection of high-quality collaborative study data.

This study has several limitations. It is a small feasibility study, and large differences were seen between treatment groups, in particular shock parameters and fibrinogen levels, which will alter treatment effects. Treatments for trauma haemorrhage are more effective when given earlier in the time course of major bleeding, as demonstrated by the CRASH-2 data [29], and it is possible that a time delay incurred in the delivery of FgC in this study may have attenuated potential clinical benefits. This study was designed to test the ability of the research teams to administer FgC rapidly, and secondary endpoints should be viewed cautiously because the study was not powered for evaluation of clinical outcomes.

## Conclusions

E-FIT 1 shows that it was not feasible to administer the study intervention within 45 minutes of admission consistently, and the pre-defined target of 90% participants was not met. Although the proportion of participants with fibrinogen levels above 2 g/L was not statistically different between arms, the rise in fibrinogen levels was greater in the active arm. There was no safety signal for thrombotic events in this study. This trial has highlighted the need for future studies to focus on the barriers to rapid delivery of concentrated fibrinogen therapies by examining each step in the trial process, including times taken to confirm eligibility and subsequent randomisation, as well as the specifics of speed of drug reconstitution and delivery.

## Abbreviations

CA5: Clot amplitude at 5 minutes
CONSORT: Consolidated Standards of Reporting Trials
DVT: Deep venous thrombosis
ED: Emergency department
FFP: Fresh frozen plasma
FgC: Fibrinogen concentrate
GCS: Glasgow Coma Scale
ISS: Injury Severity Score
MHP: Major haemorrhage protocol
MI: Myocardial infarction
PE: Pulmonary embolus
RBC: Red blood cells
RCT: Randomised controlled trial
ROTEM: Rotational thromboelastometry
SAE: Serious adverse event
TBI: Traumatic brain injury
TIC: Trauma-induced coagulopathy
TXA: Tranexamic acid
VHA: Viscoelastic haemostatic assay
